# Enhancing communication strategies in controlling neglected tropical diseases in Nigeria

**DOI:** 10.1002/puh2.100

**Published:** 2023-06-23

**Authors:** Aishat Bisoye Durojaye, Oluwakorede Joshua Adedeji, Oluwaseyi Muyiwa Egbewande, Aishat Abiodun Ibrahim, Eniola Sampson‐Oladipupo, Rashidat Onyinoyi Yusuf, Yusuf Babatunde

**Affiliations:** ^1^ Faculty of Pharmaceutical Sciences University of Ilorin Ilorin Nigeria

**Keywords:** ailments, awareness, behavioural education, communicable diseases, disease management, infections, NTD, pathogens

## Abstract

Neglected tropical diseases (NTD), a broad set of infectious diseases prevalent in tropical and subtropical environments, are widely known to affect individuals with limited resources in underserved communities. Nigeria is one of the countries with the highest cases of NTD, with an estimated 100 million people in the country at risk for at least one NTD. In Nigeria, the NTD master plan recognizes behavioural change communication as an important part of its strategy for reducing the burden of NTDs. Behavioural change communication has been proven to be significant in preventing and reducing infection, spread and re‐infection of diseases. However, poor communication strategies, lack of funds and human resources and lack of training on how to deliver behavioural change interventions are major challenges. Enhancing communication strategies will significantly help increase attention towards NTD prevention methods and acceptance of treatment interventions. It is also important to ensure that healthcare professionals are provided with adequate skills in the delivery of behavioural change interventions in the communities. This article reviews the activities to enhance communication strategies in the context settings of Nigeria.

## INTRODUCTION

Neglected tropical diseases (NTDs) are a broad set of infectious diseases prevalent in tropical and subtropical environments. NTDs affect people in rural and poor urban areas due to inadequate access to water and sanitation, as well as proximity to disease vectors and domestic animals or livestock [1]. NTDs include a diverse group of 20 conditions: Buruli ulcer, Chagas disease, dengue and chikungunya, dracunculiasis (guinea‐worm disease), echinococcosis, foodborne trematodiases, human African trypanosomiasis (sleeping sickness), leishmaniasis, leprosy (Hansen's disease), lymphatic filariasis, mycetoma, chromoblastomycosis, onchocerciasis (river blindness), rabies, scabies, schistosomiasis, soil‐transmitted helminthiases, snakebite envenoming, taeniasis/cysticercosis, trachoma and yaws [2]. The pathogens have extremely complex life cycles, infection processes and epidemiology which cause different pathologies. Their commonalities are persistence and pervasiveness in people and communities living under poverty and social exclusion [2].

In Sub‐Saharan Africa, Nigeria carries a disproportionately large burden of the global NTD disease, accounting for at least 25% of cases worldwide [1]. Partner organizations, such as the World Health Organization (WHO), United Nations Children's Fund (UNICEF)‐Nigeria, the Atlanta‐based Carter Center and its Nigerian offices in Jos, have contributed in many ways to the efforts towards the control of NTDs [3]. Other non‐governmental development organisations like the Christian Blind Mission, Sightsavers and RedAid Nigeria have been involved in the northern states of Nigeria. As a result, the most notable public health victory has been the eradication of guinea worm which causes dracunculiasis [3]. Through a mass drug administration programme, the transmissions of onchocerciasis and lymphatic filariasis have been interrupted in Plateau and Nasarawa states [4]. The elimination of trachoma has also been achieved in Gashaka, Donga and Ussa Local Government areas of Taraba state [5]. However, other health priorities have overshadowed NTD control in Nigeria. HIV/AIDS, malaria and tuberculosis receive the highest priority in disease control, even though the resources required to effectively control NTDs are relatively low compared to others [3]. In this article, we review how communication strategies can be strategized in Nigeria to ensure cost‐effective and efficient control of NTDs.

## BURDEN OF NTDS IN NIGERIA

NTDs cause approximately 534,000 deaths annually – accounting for about 10% of deaths caused by the global burden of infectious and parasitic diseases [1]. The WHO African Region accounts for almost half of the global burden of NTDs [1]. It is anticipated that Nigeria will have a significant share of the world's burden of NTDs alongside high levels of poverty by 2050. Nigeria accounts for the highest number of intestinal helminth infections, ascariasis, hookworm and trichuriasis; the highest number of schistosomiasis worldwide; the highest number of the high prevalent vector‐borne NTDs, lymphatic filariasis and onchocerciasis [5]. Nigeria has an estimated 18 million people at risk for trachoma, nearly 1.3 million people living with trichiasis, and reports the fourth‐highest number of new cases of leprosy in Africa [5]. Even though NTDs affect more than 1 billion people globally, they are still considered neglected for a lack of awareness as they mostly affect communities with poor sanitation, nutrition and quality healthcare.

NTDs burden people by impairing physical and cognitive development, depriving them of access to education and employment opportunities and limiting their productivity in the workspace. NTDs cause cycles of poverty and incur developing nations such as Nigeria billions of dollars every year in their treatment. Several efforts are needed to improve awareness about NTDs and adopt better behavioural strategies in preventing them.

## NTD PREVENTION FRAMEWORK

The leading contributor to the NTD prevention plan is the Federal Ministry of Health with the support of the Commissioner of Health, NTD programme offices of all levels of government, development partners, non‐governmental developmental organisations and the NTD steering committee [6]. In Nigeria, the first major plan was adopted in 2012, and the subsequent versions were revised in 2015 and 2022. Nigeria's prevention plan of 2012 came about after the London Declaration of NTDs which initiated an 8‐year plan to tackle NTDS in collaboration with WHO, non‐governmental organisations and pharmaceutical companies [2, 3].

In 2022, 200 of the 583 endemic local governments were assessed for lymphatic filariasis, with mass drug administration ongoing in all of these local governments [7]. One of the reasons Nigeria is still not free of NTDs is poor water sanitation. Because of the crippling impact of NTDs, the WHO devised a road map that seeks to eliminate them by 2030. With the help of the new 2023 prevention plan, Nigeria should be able to eliminate 80% of the disease burden [8].

These prevention plans from 2015 to 2022 have three key sections: situational analysis, strategic agenda and operational framework (Table 1) [6].

**TABLE 1:** The neglected tropical disease (NTD) plan 2015–2022 adopted by the Federal Ministry of Health, Nigeria.

	Key issue	Actions
Situational analysis	Nigeria has a population of over 180 million; the available healthcare facilities are not sufficient to control and meet the unending challenges in the country	Preventive chemotherapy e.g. lymphatic filariasis, onchocerciasis, schistosomiasis Case management e.g. leprosy
NTD strategic agenda	Annual mass drug administration for certain NTDs Vector control Surgery Behavioural change and communication	Increasing access to treatment Improving planning for resource mobilization Strengthening government ownership, partnership and coordination
Operational framework	Implementation of suggested interventions	Improvement of preventive chemotherapy, case management and transmission control interventions Improvement of government advocacy, coordination and partnership

The goal of the NTD programme is to decrease death and disability by putting an end to the targeted NTDs and improving the quality of life. However, Nigeria remains short of these targets.

Some reasons for this failure include the shortage of healthcare facilities. There are about 39,914 healthcare facilities for a population of 200 million, an estimated ratio of 1 hospital for every 5500 persons. Some programmes, such as schistosomiasis intervention, were delivered only to school children, whereas there is still a high proportion of the community lacking health literacy. For programme evaluations, although there has been some degree of reduction in the burden of NTDs through mass drug administration, there was poor documentation of the achievements.

## BEHAVIOURAL EDUCATION AND COMMUNICATION

Behavioural education and communication are important in empowering communities to reduce their risk of acquiring NTDs. Communication approaches through education are useful in influencing attitudes about an illness, changing behaviour to avoid or control disease, favouring disease prevention and control policies and building social norms that support healthy living. In Nigeria, the control of NTDs largely depends on the mass administration of medicines in communities and schools [2]. Although this has been a major tool for infection control, this approach cannot prevent re‐infection and necessitates the need for additional public health measures such as health education to sustain such efforts [9].

Behavioural education and communication have been proven to be significant in preventing and reducing infection, spread and re‐infection of diseases [9, 10]. For example, in Southwest Nigeria, an innovative health education game, called ‘Worms and Ladders’, was able to teach and promote good hygiene behaviour among school‐aged children and reduce the rate of infection and re‐infection of soil‐transmitted helminthiasis [10]. Therefore, the NTD prevention plans in Nigeria have placed significant emphasis on promoting behavioural change and communication.

However, there are significant gaps and challenges to implementing communication strategies in controlling NTDs. Financial constraint is one of the main barriers as funding gaps often lead to poor implementation and coverage of communication activities and messaging [11]. The other factors are the lack of human resources, poor communication networks in rural areas, lack of comprehensive integrated NTDs advocacy kits and information, education and communication materials and inadequate media publicity of NTDs [6]. Political factors, community‐level factors such as the attitudes of community stakeholders and poor understanding of the NTD programmes among policymakers and community members are also major issues in improving communication on NTDs [6].

The other public health strategies to reduce the burden of NTDs are preventive chemotherapy and transmission control, innovative and intensified disease management, vector ecology and management, veterinary public health measures and the provision of safe water, sanitation and hygiene (WASH) facilities [12]. In addition, social marketing intervention for influencing voluntary behaviours targeted at the downstream (individual), midstream (community) and upstream (government policies) factors may also be adopted.

Another strategy for behavioural education and communication is the creation of communication campaigns through television advertisements, radio spots, billboards, banners, training guides, leaflets, training videos, influencer videos and social media messages. It is, nevertheless, critical to consider the location, demographics, protective behaviours and communication channels of the target audience while developing such campaign materials.

Overall, addressing communication gaps related to NTDS in Nigeria will require bridging financial gaps, addressing human resource deficits and ensuring strong political will for implementation. It is also important to strengthen the facilitators for implementing communication interventions, such as traditional and religious institutions and the use of organised communication committees [11].

In addition to behavioural change communication, there is also a need for direct communication between patients and healthcare professionals. Most communication issues between patients and healthcare workers are due to a lack of knowledge, and feelings of embarrassment, misunderstandings and misconceptions of patients have roles in affecting communication with healthcare professionals. Healthcare professionals need to acquire adequate communication skills to establish an environment whereby patients can interact with the health personnel freely.

## CONCLUSION

An effective way to prevent NTDs is by promoting behavioural communication with at‐risk populations and those living under socioeconomic challenges. The NTD master plan in Nigeria recognizes strategizing behavioural communication change as an important component in achieving its NTD reduction targets. However, there are major challenges such as a lack of funds, human resources and adequate skills in providing behavioural change communications in the country. This highlights the need for targeted investments in building capacity and infrastructure for effective behavioural change communication to ensure that the NTD master plan in Nigeria can achieve its goals.

## AUTHOR CONTRIBUTIONS

*Supervision; writing – original draft; writing – review and editing*: Aishat Bisoye Durojaye. *Conceptualization; resources; writing – review and editing*: Oluwakorede Joshua Adedeji. *Writing – original draft; writing – review and editing*: Oluwaseyi Muyiwa Egbewande, Aishat Abiodun Ibrahim, Eniola Sampson‐Oladipupo, Rashidat Onyinoyi Yusuf. *Resources; supervision; writing – review and editing*: Yusuf Babatunde.

## CONFLICT OF INTEREST STATEMENT

All authors have no conflicts of interest.
